# Primary hemangioblastoma of the kidney with molecular analyses by next generation sequencing: a case report and review of the literature

**DOI:** 10.1186/s13000-022-01213-8

**Published:** 2022-02-27

**Authors:** Xintong Wang, George K. Haines, Meenakshi Mehrotra, Jane Houldsworth, Qiusheng Si

**Affiliations:** Department of Pathology, Molecular and Cell-Based Medicine, Icahn School of Medicine at Mount Sinai, New York, NY USA

**Keywords:** Renal hemangioblastoma, Von Hippel-Lindau disease, Next generation sequencing

## Abstract

**Background:**

Hemangioblastoma is an indolent mesenchymal tumor most frequently occurring in the central nervous system (CNS), but can also arise extraneuraxially, as part of Von Hippel-Lindau (VHL) disease or in sporadic tumors. Extraneuraxial hemangioblastomas occur outside the central nervous system. It includes tumors arising from the nervous paraneuraxial structures and visceral organs. Sporadic hemangioblastoma of the kidney, a rare subset of extraneuraxial hemangioblastomas, is an under-recognized renal neoplasm. There have been only 25 cases described to date in the English language literature. We report herein one additional sporadic tumor in a patient without VHL disease.

**Case presentation:**

A 61 year old male presenting with gross hematuria was found to have a 3.5 cm renal mass at the lateral mid to lower pole of the left kidney on computed tomography urogram. The patient underwent a partial nephrectomy for the mass. Pathological examination showed a well-circumscribed non-encapsulated tumor composed of sheets of large polygonal cells traversed by a rich vascular network. The tumor cells showed clear to eosinophilic cytoplasm and overall bland nuclei. The diagnosis of hemangioblastoma was confirmed by positive immunostaining for alpha-inhibin, S100, neuron-specific enolase, and PAX8. No significant gene mutations, including *VHL* gene and copy number changes were detected in the tumor using next generation sequencing supporting the diagnosis of sporadic renal hemangioblastoma.

**Conclusion:**

Sporadic renal hemangioblastoma is a rare subset of extraneuraxial hemangioblastomas. We report one such tumor in a patient without clinical or molecular evidence of VHL disease. The literature was reviewed to better understand the clinical, radiological, pathological, and molecular features of this neoplasm. The majority of renal hemangioblastomas showed positive immunostaining for PAX8, which supports the idea that the immunoprofiles of extraneuraxial hemangioblastomas can vary depending on sites of origin. Diagnosis of renal hemangioblastoma is challenging because of its rarity and overlapping microscopic and immunophenotypic features with other renal tumors, including clear cell renal cell carcinoma. In some cases, molecular or genetic studies may be necessary to obtain an accurate diagnosis. Since renal hemangioblastoma is clinically benign, recognition of this pathological entity is important to avoid unnecessary over-treatment.

## Background

Hemangioblastoma is an indolent tumor of mesenchymal cells that most frequently occurs in the central nervous system (CNS), mainly in the cerebellum. Most tumors are sporadic, while 20-30% occur in patients with Von-Hippel-Lindau (VHL) disease [1]. Extraneuraxial hemangioblastomas, (hemangioblastomas occurring outside the central nervous system), including tumors arising from the paraneuraxial structures, soft tissue, bone and visceral organs. Extraneural hemangioblastomas seem to be identical to the CNS hemangioblastomas morphologically and immunophenotypically, but there are certain differences. Renal hemangioblastoma is a rare subset of extraneuraxial hemangioblastomas, which usually occurs in the setting of known VHL disease. But, can also occur sporadically. To date, only 25 cases of sporadic renal hemangioblastomas have been described in the English language literature [2–16]. We report herein one such tumor in a patient without clinical or molecular evidence of VHL disease, and review the literature to better understand its clinical, radiological, pathological and molecular features. Since renal hemangioblastoma is clinically benign, a correct recognition of this pathological entity is important to avoid unnecessary clinical treatment.

## Case presentation

**Fig. 1:**
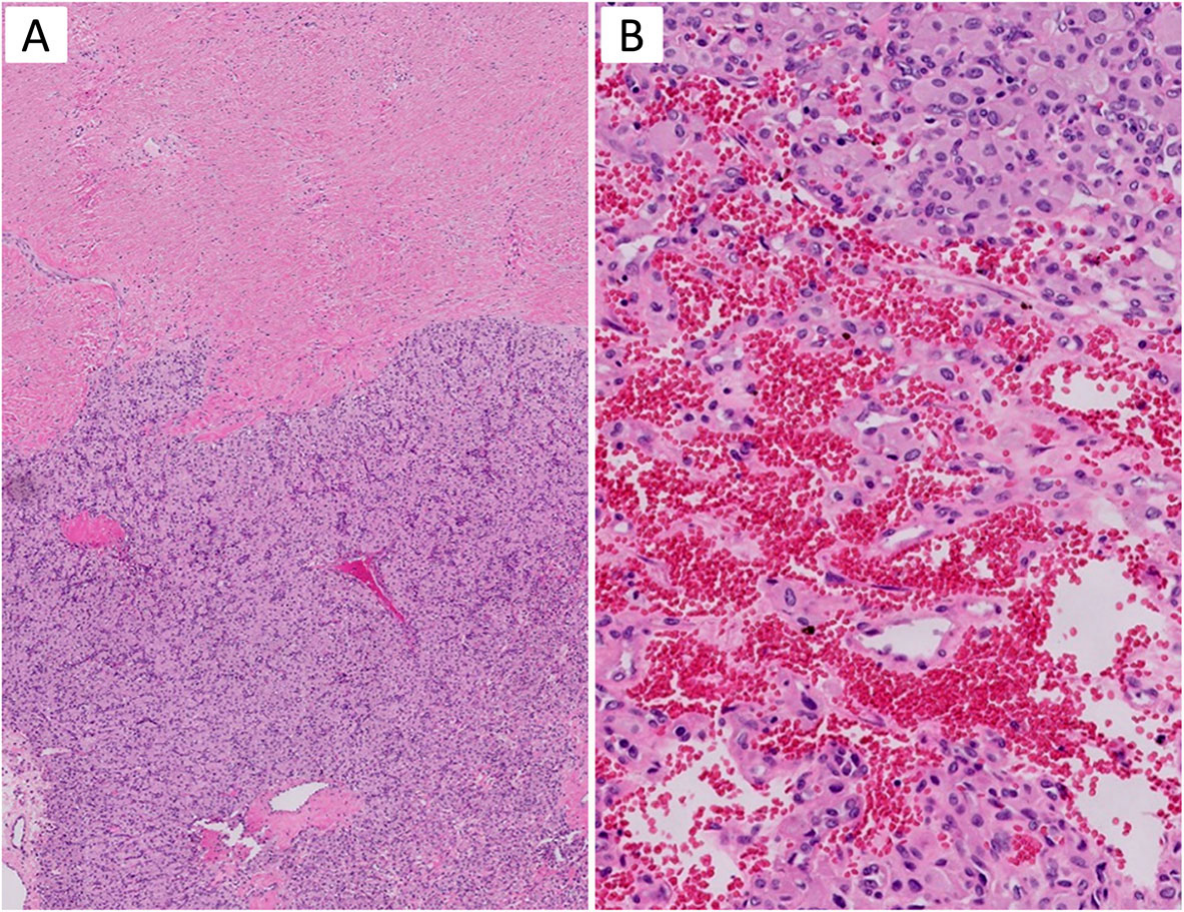
Microscopic architectural features of renal hemangioblastoma. **A** Low magnification demonstrates a solid mass divided into lobulated nodules by thick collagenous septa (H&E, × 50). **B** The tumor is traversed by arborizing thin-walled vessels (H&E. x 200)

**Fig. 2:**
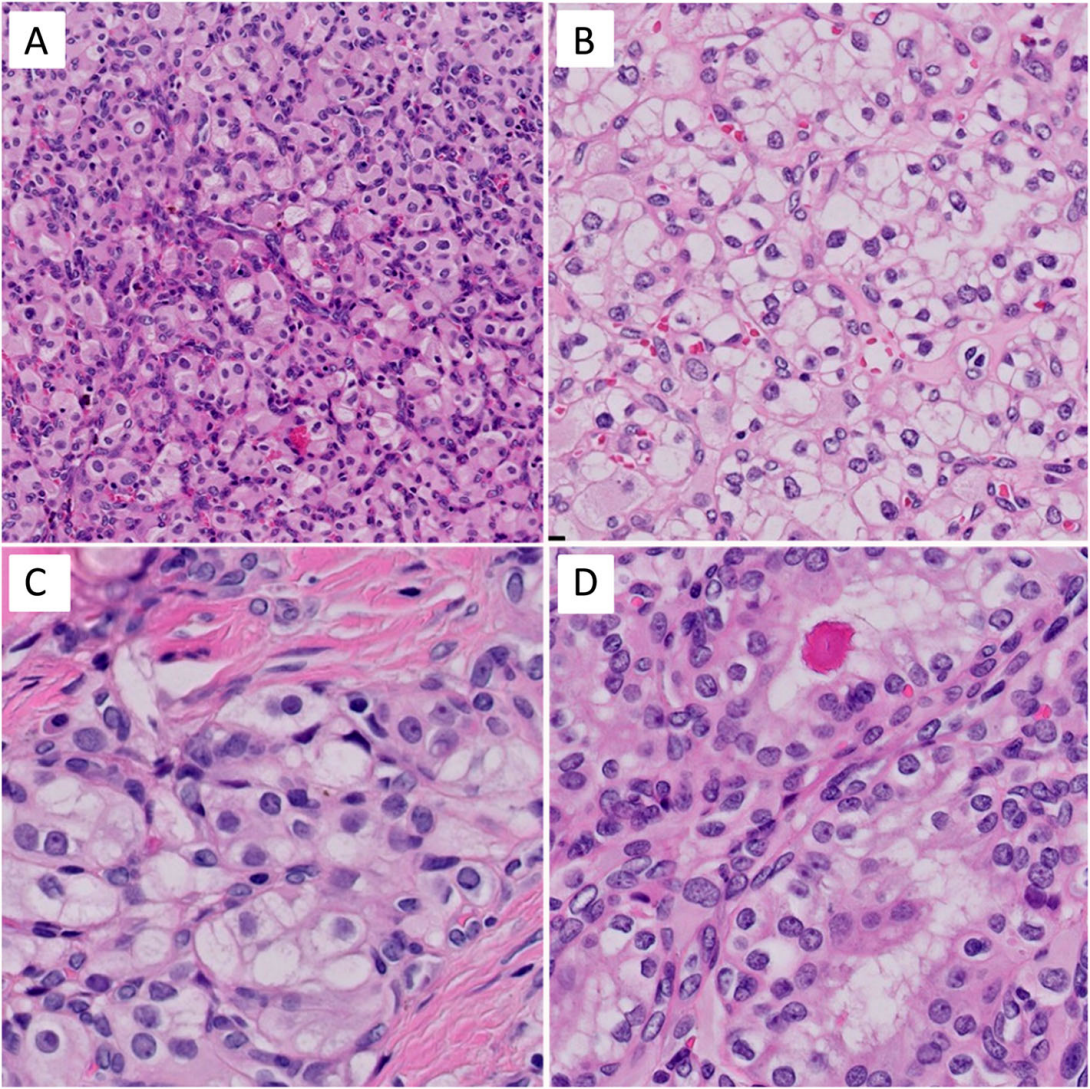
Microscopic cytologic features of renal hemangioblastoma. **A** Epithelioid tumor cells showed eosinophilic cytoplasm (H&E, x 200). **B** Few tumor cells showed clear cytoplasm (H&E, x 400). **C** Some tumor cells contain numerous well-delineated vacuoles (H&E, x 400). **D** Eosinophilic hyaline globules were found in some tumor cells (H&E, x 400)

**Fig. 3:**
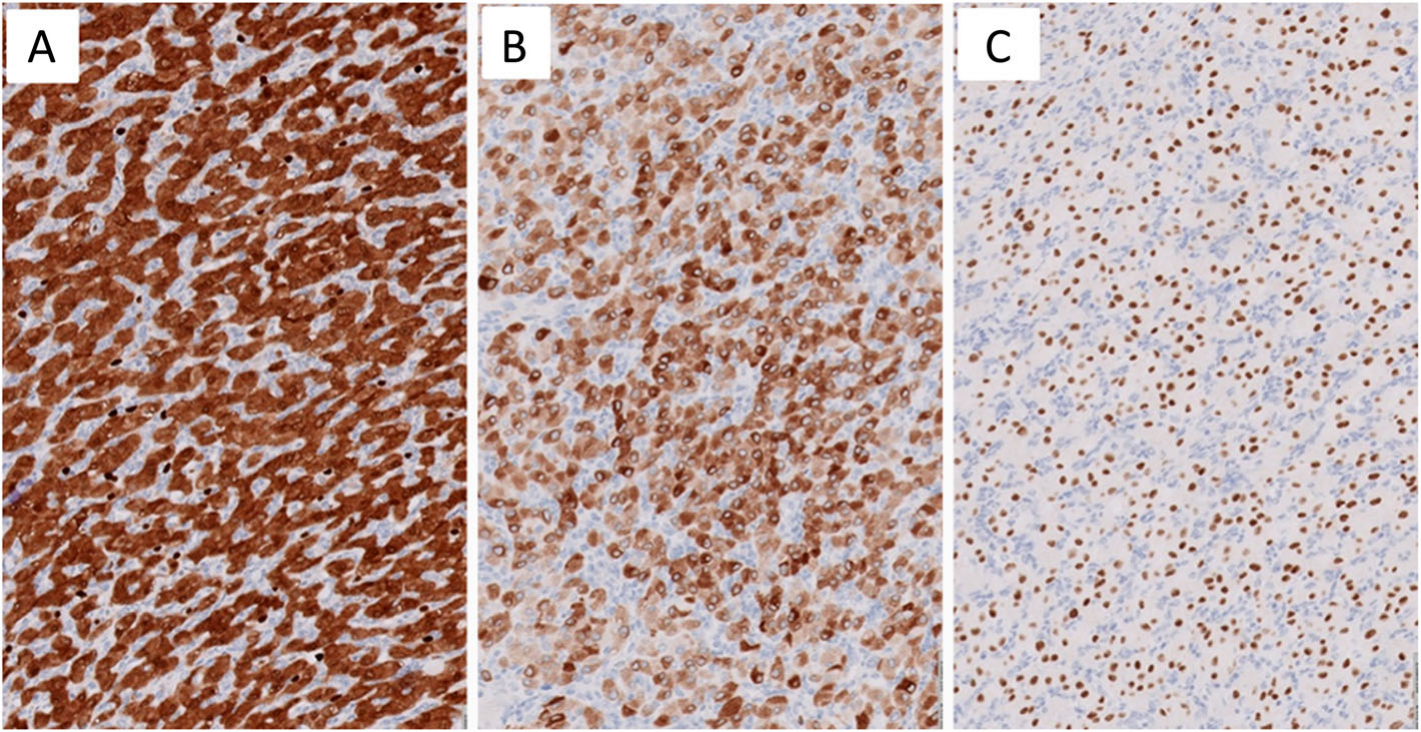
Immunohistochemical findings of renal hemangioblastoma. Tumor cells demonstrate diffuse immunoreactivity for (**A**) S100, (**B**) alpha-inhibin, and (**C**) PAX8

A 61 year-old man presented with one episode of gross hematuria without fever, flank pain, pain with urination, weight loss, or neurological symptoms. The patient had a past medical history of coronary artery disease and well-controlled hypertension. A computed tomography (CT) urogram showed a 3.5 x 2.1 x 1.3 cm, arterially enhancing mass, at the lateral mid to lower pole of the left kidney, along with a branching calculus in left lower pole renal calyces. These findings were suspicious for a renal cell carcinoma. The laboratory examination revealed a normal creatinine. There was no family history of VHL disease or neoplastic diseases. A cystoscopy was performed and revealed no tumor in the urethra, bladder or ureter. One month later, the patient underwent a laparoscopic left partial nephrectomy. Gross examination of the specimen revealed a well-circumscribed but non-encapsulated round mass, measuring 3.0 x 2.1 x 1.3 cm. Cut surfaces revealed the tumor was tan-white in color, with partial fibrosis, and mild hemorrhage. Histologically, under low power, the tumor was slightly lobulated, traversed by a prominent vascular network with thin-walled blood vessels (Fig. 1B), in a background of hyalinized and sclerotic stroma (Fig. 1A). On high power, the tumor cells were slightly variable in size, oval to polygonal in shape, and with abundant clear to eosinophilic cytoplasm (Fig. 2A and B) with occasional fine vacuoles (Fig. 2C) and eosinophilic hyaline globules (Fig. 2D). Most tumor cell nuclei were bland with inconspicuous nucleoli, although focal mild pleomorphism was present. There was no tumor necrosis or mitotic figures. Immunohistochemically, the tumor cells diffusely expressed S100 (Fig. 3A), alpha-inhibin (Fig. 3B), neuron-specific enolase (NSE), vimentin and PAX8 (Fig. 3C). AE1/AE3 showed only weak, focal cytoplasmic staining. The tumor cells were negative for epithelial membrane antigen (EMA), carbonic anhydrase 9 (CA9), CD10, KRT7, CD117, p504s, synaptophysin, chromogranin, melanin A, HMB45 and steroidogenic factor 1 (SF-1). Ki-67 stain showed very low proliferative index (<1%). Extracted genomic DNA from formalin fixed paraffin-embedded material (50% tumor cellularity) was submitted for next generation sequencing (NGS) using the Oncomine Comprehensive Plus panel (ThermoFisher) enabling the detection of variants in the full coding sequence of 227 genes and hot spots of 165 genes, and the detection of copy number variants of 333 genes, including the entire coding sequence of *VHL*. Two variants of uncertain significance were detected in *NOTCH1* (c.6061G>T, p.V2021F, variant allele frequency = 52%) and *APC* (c.7459T>C, p.S2487P, variant allele frequency = 52%), but no copy number changes were detected. Overall, the histological, immunohistochemical, and molecular findings supported the diagnosis of sporadic hemangioblastoma of the kidney. The postoperative course of the patient was uncomplicated, and there was no evidence of tumor recurrence or metastasis on follow-up 10 months after the surgery.

## Discussion

Hemangioblastoma is an uncommon, benign neoplasm that mainly occurs in the CNS, especially in the cerebellum. Extraneuraxial hemangioblastomas are rare subsets of hemangioblastomas arising outside of the CNS. There have been about 200 cases of extraneuraxial hemangioblastomas reported to date in the literature, as part of VHL disease or as sporadic tumors, and up to 140 cases were from nervous paraneuraxial structures [14]. Sporadic primary hemangioblastoma of the kidney is an even rarer neoplasm, with only 25 cases found in the English language literature (Table 1) [2–17]. Clinically, sporadic renal hemangioblastoma is a benign neoplasm, similar to other subtypes of extraneuraxial hemangioblastomas and the sporadic CNS hemangioblastoma.

**Table 1:** Reported cases of sporadic renal hemangioblastoma

Case#	Reference	Age	Gender	Tumor laterality	Site of kidney	Size of tumor (cm)	Clinical presentation	Presence of VHL disease (VHL genetic test perfromed)	Outcome
1	Nonaka et al 2007	71	F	Right	Upper pole	6.8	Asymptomatic	Not stated	ANED, 9 years
2	Ip et al 2010, case 1	58	M	Right	Mid pole	5.5	Hematuria	No	ANED, 2 years
3	Ip et al 2010, case 2	55	F	Right	Lower pole	3.5	Low back pain	No	ANED, 4 years
4	Verine et al 2011	64	M	Left	Upper pole	3.2	Asymptomatic	No	ANED, 1 year
5	Liu et al 2012	16	F	Left	Upper pole	1.2	Hematuria	No	ANED, 5 months
6	Wang et al 2012	29	M	Right	Lower pole	3.1	Asymptomatic	No	ANED, 20 months
7	Yin et al 2012	61	M	Right	Upper pole	5.3	Asymptomatic	No	ANED, 1 year
8	Jiang et al 2013	57	F	Right	Upper pole	3	Asymptomatic	No	ANED, 6 months
9	Wang et al 2013	61	M	Right	Upper pole	6.5	Asymptomatic	No	ANED, 1 year
10	Zhao et al 2013	51	F	Right	Lower pole	5.5	Abdominal pain	No	ANED, 1 year
11	Doyle et al 2014, case 1	58	M	Right	n.i.	4.5	Fevers and weight loss	No	ANED, 19 months
12	Doyle et al 2014, case 2	42	F	Left	n.i.	15.0, 4.0, 2.0 (3 tumors)	Hematuria	No	ANED, 5 months
13	Doyle et al 2014, case 3	29	M	Right	n.i.	2.7	Asymptomatic	No	ANED, 32 months
14	Kurado et al 2015	37	M	Left	Upper pole	3.6	Asymptomatic	No	n.i.
15	Wu et al 2015, case 2	48	M	Right	Lower pole	2.3	Asymptomatic	No	ANED, 42 months
16	Wu et al 2015, case 3	25	M	Left	n.i.	4.1	Asymptomatic	No	ANED, 27 months
17	Wu et al 2015, case 4	36	F	Left	n.i.	n.i.	Asymptomatic	No	ANED, 3 months
18	Wu et al 2015, case 5	57	F	Right	n.i.	n.i.	Asymptomatic	No	ANED, 5 months
19	Muscarella et al 2018, case 7	21	M	Left	n.i.	3.5	Hematuria	No	ANED, 8 years
20	Muscarella et al 2018, case 8	19	F	Right	n.i.	3	Hematuria	No	ANED, 9 years
21	Muscarella et al 2018, case 9	28	F	Right	n.i.	3.5	Asymptomatic	No	ANED, 7 years
22	Muscarella et al 2018, case 10	47	M	Right	n.i.	n.i.	n.i.	No	n.i.
23	Oberhammer et al 2019	72	F	Left	Lower pole	4.2	Asymptomatic	No	ANED, 6 months
24	He et al 2021, case 1	45	M	Left	Lower pole	3.7	Abdomenal pain	No	ANED, 5 years
25	He et al 2021, case 2	42	F	Left	n.i.	2.9	Asymptomatic	No	ANED, 3 years
26	Current case	61	M	Left	Lower pole	3	Hematuria	No	ANED, 10 months

*Abbreviation: *n.i.* no indication

As detailed in Table 1, 25 patients with sporadic renal hemangioblastomas were adults and only one was child (diagnosed at age of 16 years) [5], with the median age at diagnosis of 48 years, ranging from 16 to 71 years. Fourteen patients were male and 12 patients were female (M:F = 1.17). In 15 of 26 patients, tumors were located in the right kidney, and the upper pole was the most common site. The average tumor size was 4.2 cm (range 1.2 – 15 cm) [5, 11]. Overall, 60% of patients were asymptomatic, 24% had hematuria, 12% experienced lower back or abdominal pain, while only one patient presented with systemic symptoms, such as fever and weight loss [11]. None of the patients included in this study had VHL disease. One patient with possible VHL disease [13] was excluded from review.

Since this tumor is so rare, there are minimal descriptions of its characteristic radiological features. He et al. described peripheral nodular enhancement in the corticomedullary phase, progressive centripetal enhancement in the nephrographic and delayed phases, and sometimes complete “filling in” in the delayed phase in 2 patients with renal hemangioblastomas [16].

All reported tumors were removed surgically, with almost all (25/26) showing unilateral, unifocal distributions, with one exception [11] being a patient with 3 lesions in the left kidney (right kidney uninvolved). All lesions were confirmed as renal hemangioblastoma histologically. Macroscopically, renal hemangioblastoma displays a solid, occasionally cystic cut surface [7]. Microscopically, similarly to CNS hemangioblastoma, renal hemangioblastoma is composed of well-demarcated, large sheets of polygonal cells, with a prominent, arborizing vascular network. The tumor cells are variable in size with clear to eosinophilic cytoplasm, which commonly contains sharply delineated fine lipid vacuoles. Rhabdoid features were reported in one tumor [7]. Most of the tumors appear bland [2], but some tumors showed mild to moderate nuclear pleomorphism [3]. Mitotic figures were rare in the reported tumors.

Immunohistochemically, as detailed in Table 2, almost all of the renal hemangioblastomas demonstrated diffuse positivity for alpha-inhibin (24/26), NSE (23/23), S100 protein (25/25), and vimentin (19/21), and negativity for neuroendocrine markers (synaptophysin, chromogranin A), melanocytic markers (HMB45, melan-A), endothelial markers (CD31, CD34), and mesothelial markers (calretinin, WT-1). In 3 of 21 reported cases, tumor cells expressed focal, patchy positivity for AE1/AE3 [10, 16], including the present tumor. The majority of renal hemangioblastomas exhibited no immunoreaction for muscle, such as desmin [2–4], but two tumors were noted to have focal expression of smooth muscle actin [2, 15]. CD10 was reported to be positive in 7/16 tumors [7, 8, 10, 14, 15]. EMA was positive in 5/15 tumors [4, 7, 8, 14, 15] and CA9 was positive in 3/4 tumors [4, 8, 12].

**Table 2:** Immunohistochemical findings of reported cases of sporadic renal hemangioblastoma

Case#	Reference	S100	Inhibin	Vimentin	NSE	PAX8	PAX2	CD10	WT1	Calretinin	Pan-cytokeratin	CK7	EMA	CAIX	SMA	Desmin	CD31	CD34	Synaptophysin	Chromogranin	Melanin A	HMB45	CD117
1	Nonaka et al 2007	+	+	+	n.i.	n.i.	n.i.	n.i.	-	-	-	n.i.	-	n.i.	+	-	-	-	n.i.	-	n.i.	-	n.i.
2	Ip et al 2010, case 1	+	+	n.i.	+	n.i.	n.i.	n.i.	n.i.	-	-	n.i.	n.i.	n.i.	-	-	n.i.	-	-	-	-	-	n.i.
3	Ip et al 2010, case 2	+	+	n.i.	+	n.i.	n.i.	n.i.	n.i.	-	-	n.i.	n.i.	n.i.	-	-	n.i.	-	-	-	-	-	n.i.
4	Verine et al 2011	+	+	+	+	n.i.	n.i.	-	n.i.	-	-	n.i.	+	+	-	-	n.i.	-	-	-	-	-	n.i.
5	Liu et al 2012	+	+	+	+	n.i.	n.i.	-	n.i.	-	-	n.i.	-	n.i.	-	-	-	-	-	-	-	-	n.i.
6	Wang et al 2012	+	+	+	+	n.i.	n.i.	-	n.i.	n.i.	-	n.i.	n.i.	n.i.	-	n.i.	n.i.	-	n.i.	n.i.	-	-	n.i.
7	Yin et al 2012	+	+	+	+	n.i.	n.i.	Focal+	n.i.	-	-	n.i.	Focal+	n.i.	-	-	n.i.	-	n.i.	-	-	-	n.i.
8	Jiang et al 2013	+	+	n.i.	+	n.i.	+	Focal+	n.i.	n.i.	-	n.i.	n.i.	+	-	n.i.	n.i.	n.i.	n.i.	n.i.	-	-	n.i.
9	Wang et al 2013	+	+	n.i.	+	n.i.	n.i.	n.i.	n.i.	n.i.	n.i.	n.i.	n.i.	n.i.	n.i.	n.i.	n.i.	n.i.	n.i.	n.i.	n.i.	n.i.	n.i.
10	Zhao et al 2013	+	+	+	+	+	n.i.	+	n.i.	n.i.	Focal+	-	Focal+	n.i.	-	-	-	-	-	-	-	-	-
11	Doyle et al 2014,case 1	+	+	+	+	Weak+	n.i.	n.i.	-	n.i.	-	n.i.	-	n.i.	n.i.	n.i.	n.i.	n.i.	n.i.	n.i.	n.i.	n.i.	n.i.
12	Doyle et al 2014,case 2	+	+	+	+	n.i.	n.i.	n.i.	-	n.i.	-	n.i.	-	n.i.	n.i.	n.i.	n.i.	n.i.	n.i.	n.i.	n.i.	n.i.	n.i.
13	Doyle et al 2014,case 3	+	+	+	+	n.i.	n.i.	n.i.	-	n.i.	-	n.i.	-	n.i.	n.i.	n.i.	n.i.	n.i.	n.i.	n.i.	n.i.	n.i.	n.i.
14	Kurado et al 2015	+	+	+	n.i.	+	+	-	n.i.	n.i.	-	n.i.	n.i.	+	-	n.i.	n.i.	n.i.	n.i.	n.i.	-		n.i.
15	Wu et al 2015, case 2	+	-	+	n.i.	-	n.i.	-	n.i.	n.i.	-	-	-	n.i.	n.i.	n.i.	n.i.	n.i.	n.i.	n.i.	n.i.		n.i.
16	Wu et al 2015, case 3	+	+	-	+	-	n.i.	-	n.i.	n.i.	-	n.i.	n.i.	n.i.	n.i.	n.i.	n.i.	n.i.	n.i.	n.i.	n.i.	-	n.i.
17	Wu et al 2015, case 4	+	-	-	+	-	n.i.	-	n.i.	n.i.	-	n.i.	n.i.	n.i.	n.i.	n.i.	n.i.	n.i.	n.i.	n.i.	n.i.	-	n.i.
18	Wu et al 2015, case 5	+	+	+	+	-	n.i.	+	n.i.	n.i.	-	n.i.	n.i.	n.i.	n.i.	n.i.	n.i.	n.i.	n.i.	n.i.	n.i.	-	n.i.
19	Muscarella et al 2018,case 7	+	+	+	+	+	n.i.	+	n.i.	n.i.	n.i.	n.i.	-	n.i.	-	n.i.	n.i.	-	-	-	n.i.	-	-
20	Muscarella et al 2018,case 8	+	+	+	+	-	n.i.	+	n.i.	n.i.	n.i.	n.i.	-	n.i.	-	n.i.	n.i.	-	-	-	n.i.	-	-
21	Muscarella et al 2018,case 9	+	+	+	+	+	n.i.	-	n.i.	n.i.	n.i.	n.i.	+	n.i.	-	n.i.	n.i.	-	-	-	n.i.	-	-
22	Muscarella et al 2018,case 10	+	+	+	+	-	n.i.	n.i.	n.i.	n.i.	n.i.	n.i.	-	n.i.	-	n.i.	n.i.	-	-	-	n.i.	-	-
23	Oberhammer et al 2019	+	+	+	+	+	n.i.	+	+	n.i.	-	-	+	n.i.	+	-	n.i.	-	n.i.	n.i.	-	-	n.i.
24	He et al 2021, case 1	n.i.	+	+	+	n.i.	n.i.	n.i.	n.i.	n.i.	Focal+	n.i.	n.i.	n.i.	n.i.	n.i.	-	-	n.i.	n.i.	n.i.		n.i.
25	He et al 2021, case 2	+	+	n.i.	+	+	n.i.	n.i.	n.i.	n.i.	-	n.i.	n.i.	n.i.	n.i.	n.i.	-	-	n.i.	n.i.	n.i.		n.i.
26	Current case	+	+	+	+	+	n.i.	-	n.i.	n.i.	Focal+	-	-	-	n.i.	n.i.	n.i.	n.i.	-	-	-	-	-

*Abbreviation: *n.i.* no indication

Including the present case, the diffuse and strong nuclear positivity for PAX8 was observed in 8/14 tumors [10–12, 14–16], and PAX2 was positive in 2 reported tumors [8, 12]. PAX8 and PAX2 are cell lineage specific transcription factors that play a crucial role in the organogenesis of the kidney [17]. Both factors are expressed in normal kidneys as well as in many renal epithelial neoplasms such as renal cell carcinoma (RCC). Zhao et al, has put forward a hypothesis suggesting that the immunoprofile of extraneuraxial hemangioblastomas can vary with different sites of origin [10]. With the findings of positivity for PAX8 and/or PAX2 in the above-mentioned 9 tumors, we agree with the hypothesis that renal hemangioblastomas are capable of expressing kidney-specific antigens.

Renal hemangioblastoma is likely to be an under-recognized tumor of kidney due to its rarity. This indolent neoplasm can be mistaken for various malignancies, including clear cell RCC or epithelioid angiomyolipoma. Clear cell RCC share similar morphological characteristics with renal hemangioblastomas, such as a clear cytoplasm and a prominent vascular network. The most useful feature to differentiate clear cell RCC from renal hemangioblastoma is the absence of fine cytoplasmic lipid vacuoles, which is predominantly present in renal hemangioblastoma. Immunohistochemically, clear cell RCC is usually positive for AE1/AE3, EMA, CA9, CD10 and PAX8, but negative for alpha-inhibin, S100, and NSE. However, to add to the confusion, Montironi et al [18] reported clear cell RCCs in 2 patients with 60-70% of tumor cells showing hemangioblastoma-like features. In those reported cases, the tumor cells expressed alpha-inhibin and S100 in the hemangioblastoma-like part only (not in the clear cell RCC part), but PAX8, CD10, and RCC were positive in both components of the tumor. After carefully reviewing the histologic morphology in our present case, we have found ~10% of tumor cells showing clear cytoplasm, but all tumor cells stained consistently for S100, alpha-inhibin, NSE, Vimentin, and negative for CD10 and CA9. These findings supported a diagnosis of renal hemangioblastoma. Another major mimicker of renal hemangioblastoma is epithelioid angiomyolipoma, which also possesses sheets of polygonal cells with an abundant cytoplasm and a rich vascular network. The key feature to differentiate renal hemangioblastoma from epithelioid angiomyolipomas is that epithelioid angiomyolipomas usually show reticulated cytoplasm instead of a lipid containing vacuolated cytoplasm. Immunohistochemically, epithelioid angiomyolipomas are usually HMB45 positive, melan-A positive, but alpha-inhibin negative. Other differential diagnoses include renal oncocytoma and intrarenal ectopic adrenal tissue. Oncocytoma shares similar morphological characteristics with hemangioblastomas, such as eosinophilic cytoplasm and eosinophilic hyaline globules, but fine cytoplasmic lipid vacuoles are absent in oncocytoma. Immunohistochemically, oncocytoma is usually positive for EMA, CD117, and PAX8, but negative for CD10, alpha-inhibin and vimentin. Intrarenal ectopic adrenal tissue can be found in 6% of the general population. It commonly composed of sheets or glands of cells with distinct cell borders and abundant foamy cytoplasm, traversed by interspersed blood vessels in a sinusoidal pattern, which features are similar to the hemangioblastoma. Although adrenal cortical cells are positive for a-inhibin, they also show immunoreactivity for other adrenocortical markers like melan-A and SF-1, but are negative for PAX8, S100 and vimentin.

In some reported cases, molecular genetic studies were performed, including polymerase chain reaction (PCR) analysis for exons of *VHL* gene (tested in 6 studies, 9 patients) [3, 6, 8, 12, 14, 15], loss of heterozygosity (LOH) analysis for chromosome 3p genes (tested in 2 studies, 5 patients) [12, 14], and fluorescence in situ hybridization (FISH) for chromosome 3p deletion (tested in 1 study, 1 patient) [8]. Overall, no *VHL* gene mutation, loss of heterozygosity (LOH) of chromosome 3p or chromosome 3p deletion were detected in the reported 9 tumors. We agree with the hypothesis put forward by Muscarella et al. that 1) the genetic changes may be localized to the intronic or regulatory regions of *VHL* gene; 2) the genetic anomaly may involve other genes, which interplay with *VHL* gene expression; 3) alternative tumor genetic mechanisms [14]. Since NGS can afford a comprehensive molecular profiling approach, we submitted the tumor for NGS that interrogated DNA alterations in over 400 genes, with no significant alterations detected. At the time of our study, this is the first time that NGS analysis has been described in sporadic renal hemangioblastoma. However, no significant gene mutations, including *VHL* gene were detected in our tumor. Two variants of uncertain significance were detected in *NOTCH1* and *APC*, but no copy number changes were detected. It may require a large cohort study to address the significance of these two variants in renal hemangioblastoma. Therefore, the tumor genetic mechanism of renal hemangioblastoma, at least in our tumor, remained unclear.

## Conclusion

Sporadic renal hemangioblastoma is a rare subset of extraneuraxial hemangioblastomas. We report herein one such tumor in a patient without clinical or molecular evidence of VHL disease, and reviewed the literature to better understand the clinical, radiological, pathologic and molecular features of this neoplasm. From our review cases and the present case, we have found that the majority of renal hemangioblastomas showed positive immunostaining for PAX8, which supports the idea that the immunoprofile of extraneuraxial hemangioblastomas can vary depending on sites of origin. Diagnosis of renal hemangioblastoma is challenging because of its rarity and overlapping microscopic and immunophenotypical features with renal cell tumors (PAX8+ and CD10+), especially clear cell renal cell carcinoma. However, accurate diagnosis is necessary, since renal hemangioblastoma is clinically benign diagnosis, and a correct recognition of this pathological entity is important to avoid unnecessary treatment.

## Data Availability

The dataset supporting the conclusions of this article is included within the article.

## Abbreviations

CA9: carbonic anhydrase 9
CNS: central nervous system
CT: computed tomography
EMA: epithelial membrane antigen
NGS: next generation sequencing
NSE: neuron-specific enolase
RCC: renal cell carcinoma
VHL: Von Hippel-Lindau
